# Levonorgestrel-releasing intrauterine device treatment in heavy menstrual bleeding: Correlation with endometrial pathology and quality of life

**DOI:** 10.1371/journal.pone.0338480

**Published:** 2025-12-11

**Authors:** Selma Atiye Kolcu, Ebru Kara, Ayça Kubat Küçükyurt, Elif Yıldız, Arzu Çetin

**Affiliations:** Department of Gynecology and Obstetrics, İstanbul Training And Research Hospital, İstanbul, Turkey; Tarbiat Modares University, IRAN, ISLAMIC REPUBLIC OF

## Abstract

The aim of the study was to compare the effects of 52 mg Levonorgestrel-releasing intrauterine device (LNG-IUD), which is frequently used in patients with heavy menstrual bleeding, on quality of life according to benign endometrial pathology patterns. The study was designed retrospectively.143 patients between the ages of 28 and 54, who underwent endometrial sampling due to heavy menstrual bleeding and then the 52 mg LNG-IUD was placed, were included in the study. The most commonly observed results in endometrial pathology were divided into 2 groups: normal cycle patterns and hormonal imbalance patterns. Change in quality of life after LNG-IUD insertion was assessed using the Menorrhagia Multiattribute Scale (MMAS), the 36-Item Short Form Health Survey (SF-36), and the Beck Depression Inventory. In both groups, a significant increase was observed in all parameters of the SF-36 quality of life scale and the MMAS total score after LNG-IUD use compared to the pre-IUD period (p < 0.05) (MMAS increased from 51.9 ± 27.8% to 83.8 ± 26.2% at 6 months), and a significant decrease was observed in the Beck Depression Inventory score and depression rate (p < 0.05). However, there was no significant difference in the changes in quality of life questionnaires before and after LNG-IUD use between the groups (p > 0.05). The LNG-IUD provides substantial improvements in bleeding control and quality of life among women with heavy menstrual bleeding, and these effects are not influenced by endometrial pathology results.

## Introduction

Heavy menstrual bleeding is a common issue that can significantly affect the lives of women of reproductive age and defined as losing more than 80 ml of menstrual blood per cycle [1]. The prevalence of heavy menstrual bleeding in reproductive-age women, based on objective measurements, ranges from 9% to 14%, but studies that rely on subjective assessments have reported rates as high as 20% to 52% [2,3].

Since heavy menstrual bleeding is a subjective evaluation, it has been stated in various international guidelines that the diagnosis should not be based solely on objective measurements, and that it would be more accurate to evaluate it as excessive menstrual blood loss that affects women’s physical, emotional, social and material quality of life [4–6]. To diagnose underlying causes, imaging techniques such as pelvic or transvaginal ultrasound and hysteroscopy are utilized, along with tissue sampling. Endometrial biopsy has high sensitivity for detecting benign, premalignant and malignant intrauterine pathologies [7,8]. In patients with heavy menstrual bleeding, the most common endometrial sampling results reveal a normal cyclic endometrium (proliferative, secretory and shedding phases) (28.6–56.8%), while the most frequently observed pathology, regardless of age group, is hormonal imbalance patterns (7.5–19.5%). Within the hormonal imbalance pattern, conditions such as irregular proliferative endometrium (7.5–19.5%), stromal and glandular destructive non-secretory endometrium, luteal phase defect, and medication effects can be identified. Irregular proliferative endometrium represents an exaggerated proliferative phase indicative of chronic anovulation, especially during the perimenopausal years. Other causes include pregnancy complications, endometrial polyps, endometrial hyperplasia, carcinomas, and chronic endometritis [9–11].

Heavy menstrual bleeding can be treated using medical or surgical methods, depending on its etiology [12]. Medical treatment including hormonal drugs and other pharmacological approaches is widely used. One such option is the 52 mg Levonorgestrel-releasing intrauterine device (LNG-IUD), which is recommended as the best first-line treatment for patients with heavy menstrual bleeding who have no additional pathologies or have fibroids smaller than 3 centimeters or adenomyosis [13]. Various studies have demonstrated the efficacy of the LNG-IUD compared to other systemic medications and surgical options, showing that it not only effectively manages symptoms but also contributes to better quality of life [14,15].

There are studies in the literature evaluating the efficacy of the LNG-IUS according to the underlying medical pathology. Several studies have investigated the effectiveness of the LNG-IUS in different types and severities of adenomyosis, reporting variable results [16,17]. Similarly, in women with uterine fibroids, the LNG-IUS has been shown to be effective in reducing heavy menstrual bleeding, although higher expulsion rates have been reported [18]. In addition, the LNG-IUS has been found to thin the endometrium and to be highly effective in cases of localized endometrial pathology [19]. However, no study has been found comparing the effects of benign endometrial pathology results on the quality of life after LNG-IUS treatment. Therefore, the aim of the present study was to evaluate whether endometrial sampling results have an impact on the treatment outcomes and quality of life in patients with heavy menstrual bleeding using the LNG-IUS.

## Materials and methods

**Fig 1:**
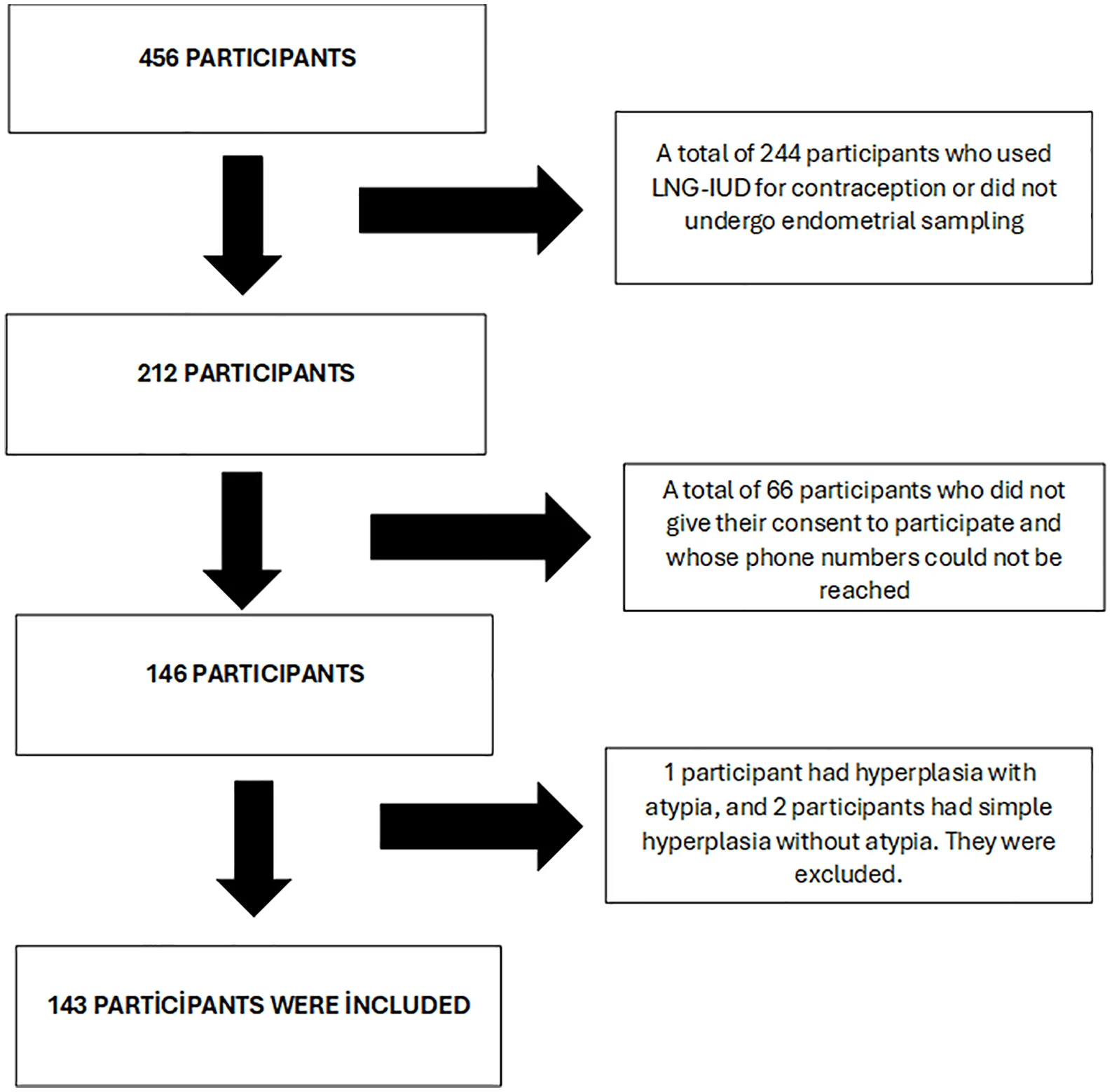
Flow diagram showing details of patients included in the study.

The study is a retrospective cohort study conducted at the Training and Research Hospital. All patients who underwent a 52 mg LNG-IUD (Mirena® Bayer, Whippany, N J 07981, Leverkusen, Germania) insertion between January 1, 2023, and January 30, 2024 were screened from the hospital system, and a total of 456 patients were identified. Of these patients, 244 were excluded from the study because endometrial pathology sampling was not performed or because LNG-IUD insertion was performed for contraceptive purposes. Of the 212 patients who underwent endometrial sampling, 66 were excluded because their phone numbers were unavailable or they refused to participate in the study. 1 patient with hyperplasia with atypia and 2 patients with simple hyperplasia without atypia were excluded. A total of 143 patients aged 28–54 years who underwent endometrial sampling due to heavy menstrual bleeding and subsequently underwent LNG-IUD insertion were included in the study (Fig 1). Medical records of the participants were reviewed between 26.07.2024 and 10.12.2024.

Patient demographic data was extracted from hospital records. They were then contacted by phone and given a retrospective questionnaire about their clinical status before and six months after LNG-IUD placement. The results were documented in writing on the questionnaire forms. Menstrual pattern 6 months after LNG-IUD insertion was noted according to The International Federation of Obstetrics and Gynecology (FIGO) current classification of abnormal uterine bleeding [20].

All endometrial sampling was performed before The LNG-IUD insertion due to heavy menstrual bleeding. The LNG-IUD was inserted at any time when the researcher is reasonably certain that the participant is not pregnant. After the device was inserted, transvaginal ultrasound was used to confirm correct placement. Additionally, blood hemoglobin and hematocrit levels were evaluated on the same day.

Those who declined to participate or did not give consent to join the study were excluded (Fig 1). Anticoagulant users, individuals with hereditary coagulation disorders, those with uterine fibroids larger than 3 cm, patients who did not undergo endometrial sampling, patients without heavy menstrual bleeding who received an LNG-IUD for contraception, and those with bleeding due to cervical or vaginal etiologies were excluded from the study. Also 3 patients who underwent LNG-IUD but had endometrial pathologies such as atypical (1 case) or simple endometrial hyperplasia (2 cases) were excluded from the study.

Endometrial pathology results of patients with LNG-IUD insertion were obtained from the hospital’s electronic system. The most common endouterine sampling results for heavy menstrual bleeding were normal cycle patterns (26.7%) and hormonal imbalance patterns (30.5%). The next most common endometrial pathology result was endometrial polyps (24.3%) [21]. In our study, patients were divided into two groups as normal cycle patterns and hormonal imbalance patterns to evaluate the outcome of LNG-IUD treatment. Because endometrial polyps are treated surgically, LNG-IUDs are not included in the study because they are not administered. Patients whose endometrial pathology revealed proliferative, secretory, and shedding phases were included in the normal cycle pattern group. Patients whose endometrial pathology revealed irregular proliferative endometrium, hormonal effects, or anovulatory endometrium were included in the hormonal imbalance pattern group.

In this study, three assessment tools were utilized: MMAS questionnaire, the Medical Outcomes Study 36-Item Short Form Health Survey (MOS SF-36), and the Beck Depression Inventory. In 1998, Shaw and colleagues designed the Menorrhagia Multiattribute Scale (MMAS), which is widely used today for the subjective assessment of menorrhagia-related quality of life. The MMAS has demonstrated high construct validity and reliability, demonstrating strong internal consistency and retest reliability, and it also measures treatment outcomes. The MMAS questionnaire assesses the impact of abnormal uterine bleeding on six different domains of life, including practical difficulties, social life, psychological health, physical health and well-being, daily routines, and family relationships [22,23]. The MMAS questionnaire was translated into the local language by the researchers conducting this study and administered to patients during telephone interviews, with responses recorded by the gynecologists.

The SF-36 questionnaire, a reliable and well-validated tool for assessing overall well-being, consists of 36 items that measure 8 scales: vitality, physical functioning, bodily pain, general health perception, physical role functioning, emotional role functioning, social role functioning, and mental health [24].

The Beck Depression Inventory is a 21-item scale used to assess a patient’s psychological health. Each question has four response options. The total score from the patient’s responses is categorized as follows: 0–9 indicates normal, 10–18 indicates mild depression, 19–29 indicates moderate depression, and 30–63 indicates severe depression.

The study was conducted in accordance with the Declaration of Helsinki. Approval was received from the Ethics Committee with decision number 368 dated December 22, 2023.

Statistical analysis: Descriptive statistics for the data included mean, standard deviation, median, minimum, maximum, frequency, and percentage values. The Kolmogorov–Smirnov and Shapiro–Wilk tests were applied to continuous variables to assess whether they followed a normal distribution before selecting appropriate parametric or non-parametric statistical tests. For normally distributed quantitative independent data, the independent samples t-test was used, while the Mann-Whitney U test was employed for non-normally distributed quantitative independent data. The Wilcoxon test was applied for dependent quantitative data, and the chi-square test was used for qualitative independent data. Analyses were conducted using SPSS version 28.0.

## Results

The demographic data of the 143 patients included in the study are presented in Table 1. Histopathological examination showed a variety of patterns consisting of normal cyclical patterns showing proliferative, secretory, and shedding phases in 93 of the 143 total cases. (Table 1).

**Table 1:** Demographic data of patients included in the study.

		Min-Max	Md	Mean±SD	n (%)
Age		28.0–54.0	43.0		
Comorbidity	No				103 (72.0%)
	Yes				40 (28.0%)
	Hypothyroidism				21 (14.7%)
	Hypertension				7 (4.9%)
	Other Diseases				20 (14.0%)
Gravida		0.0–7.0	3.0		
Parity		0.0–5.0	3.0		
Pre - LNG-IUD^†^ Hg(g/dL)^‡^				11.4 ± 1.7	
Post - LNG-IUD Bleeding Pattern	Normal				78 (54.5%)
	Heavy Menstrual Bleeding				38 (26.6%)
	Amenorrhea				15 (10.5%)
	Intermenstrual Bleeding				12 (8.4%)
Was the LNG-IUD Removed? (expulsion + elective)	No				119 (83.2%)
	Yes				24 (16.8%)
Hysterectomy	No				131 (91.6%)
	Yes				12 (8.4%)
Endometrial Sampling Result	Normal Cycle Pattern				93 (65.0%)
	Hormonal Imbalance Pattern				50 (35.0%)
n					143 (100%)

† LNG-IUD: The Levonorgestrel Releasing Intrauterine Device ‡Hg: Hemoglobin n: Total Number of Cases.

After the application of the LNG-IUD, all parameters of the SF-36 quality of life scale and the total MMAS score showed a significant increase compared to before the LNG-IUD insertion (p < 0.05) (Table 2). Additionally, the Beck Depression Inventory score and depression rate significantly decreased after the LNG-IUD application compared to before (p < 0.05) (Table 2)

**Table 2:** Changes in SF-36, MMAS questionnaires and Beck Depression Inventory after LNG-IUD insertion.

		Pre - LNG-IUD^†^			Post - LNG-IUD				
		Mean± SD	n(%)	Md	Mean± SD	n(%)	Md	p	
Depression	None	134 (93.7%)			140 (97.9%)			0.031	N
	Mild	9 (6.3%)			3 (2.1%)				
Beck Depression Inventory				2.0			0.0	0.000	W
MMAS^‡^ Total Score		51.9 ± 27.8		47.7	83.8 ± 26.2		100.0	0.000	W
SF-36^§^ Quality of Life Scale									
Physical Functioning		78.6 ± 21.6		85.0	92.3 ± 17.4		100.0	0.000	W
Role Physical		29.0 ± 43.4		0.0	82.3 ± 37.0		100.0	0.000	W
Role Emotional		29.4 ± 44.5		0.0	80.6 ± 38.6		100.0	0.000	W
Vitality		48.6 ± 19.6		45.0	60.4 ± 18.3		60.0	0.000	W
Mental Health		67.0 ± 16.3		68.0	70.6 ± 15.3		72.0	0.000	W
Social Functioning		67.5 ± 23.3		67.5	82.6 ± 21.8		100.0	0.000	W
Bodily Pain		76.0 ± 27.2		87.5	87.3 ± 22.5		100.0	0.000	W
General Health		45.1 ± 20.0		45.0	58.6 ± 17.5		65.0	0.000	W

Md: Median ^w^ Wilcoxon test ^N^ MC Nemar test †: LNG-IUD: The Levonorgestrel Releasing Intrauterine Device ‡: MMAS: Menorrhagia Multi-Attribute Scale §: SF-36: 36-Item Short Form Health Survey.

In the group with a normal cyclic pattern from endometrial sampling, the patients’ age was significantly lower than that of the group with a hormonal imbalance pattern (p < 0.05) (Table 3).

**Table 3:** Based on endometrial sampling results; Comparison of changes in bleeding pattern, treatment continuation rate, and Beck depression inventory after LNG-IUD insertion.

	Endometrial Sampling Result						
	Normal Cycle Pattern (n:93)			Hormonal Imbalance Pattern (n:50)			
		n (%)	Md	n (%)	Md	p	
Age			41.0		45.0	0.001	m
Comorbidity	No	66 (71.0%)		37 (74.0%)		0.700	X²
	Yes	27 (29.0%)		13 (26.0%)			
Hypothyroidism		16 (17.2%)		5 (10.0%)		0.246	X²
Hypertension		4 (4.3%)		3 (6.0%)		0.653	X²
Others Diseases		11 (11.8%)		9 (18.0%)		0.310	X²
Gravida			3.0		3.0	0.901	m
Parity			2.0		3.0	0.927	m
Pre-LNG IUD)^†^ Hg(g/dL)^‡^			11.7		11.7	0.749	m
Post LNG-IUD Bleeding Pattern							
Normal		54 (58.1%)		24 (48.0%)		0.093	X²
Heavy Menstrual Bleeding		24 (25.8%)		14 (28.0%)			
Amenorrhea		11 (11.8%)		4 (8.0%)			
Intermenstrual Bleeding		4 (4.3%)		8 (16.0%)			
Was the LNG-IUD Removed? (expulsion+ elective)	No	79 (84.9%)		40 (80.0%)		0.450	X²
	Yes	14 (15.1%)		10 (20.0%)			
Hysterectomy	No	88 (94.6%)		43 (86.0%)		0.076	X²
	Yes	5 (5.4%)		7 (14.0%)			
Beck Depression Inventory Score							
Pre LNG-IUD			2.0		2.0	0.559	m
Post LNG-IUD			0.0		0.0	0.518	m
Depression Rate							
Pre LNG-IUD	None	86 (92.5%)		48 (96.0%)		0.408	X²
	Mild	7 (7.5%)		2 (4.0%)			
Post LNG-IUD	None	91 (97.8%)		49 (98.0%)		1.000	X²
	Mild	2 (2.2%)		1 (2.0%)			

Md: Median ^m^ Mann-whitney u test ^X²^ Ki-kare test (Fischer test) †: LNG-IUD: The Levonorgestrel Releasing Intrauterine Device ‡: Hg: Hemoglobin.

There were no significant differences between the groups with normal cyclic pattern and hormonal imbalance pattern regarding gravida, parity, the rate of having any additional diseases, hypothyroidism, hypertension, other comorbidities, and hemoglobin levels before LNG-IUD insertion (p > 0.05) (Table 3).

There were no significant differences between the groups with normal cyclic pattern and hormonal imbalance pattern regarding bleeding pattern after LNG-IUD insertion, the rate of LNG-IUD removal, and the rate of hysterectomy (p > 0.05) (Table 3). Additionally, the depression rate and Beck Depression Inventory scores did not show significant differences between the groups (p > 0.05) (Table 3).

When comparing the groups with normal cyclic pattern and hormonal imbalance pattern based on endometrial pathology, there were no significant differences in all parameters of the SF-36 questionnaire and the total MMAS score before and after LNG-IUD insertion (p > 0.05) (Table 4).

**Table 4:** Comparison of changes in SF-S6 and MMAS total scores before and after LNG-IUD insertion according to endometrial sampling results.

	Endometrial Sampling Result					
	Normal Cycle Pattern (n:93)		Hormonal Imbalance Pattern (n:50)			
	Q1–Q3	Md	Q1–Q3	Md	p	
SF-36^†^ Physical Functioning Score						
Pre LNG-IUD^‡^	65,0–95,0	85.0	70,0–100,0	90.0	0.267	m
Post LNG-IUD	100,0–100,0	100.0	95,0–100,0	100.0	0.715	m
SF-36 Role Physical						
Pre LNG-IUD	0,0–75,0	0.0	0,0–100,0	0.0	0.356	m
Post LNG-IUD	100.0–100,0	100.0	100,0–100,0	100.0	0.406	m
SF-36 Role Emotional						
Pre LNG-IUD	0,0–66,4	0.0	0,0–100,0	0.0	0.434	m
Post LNG-IUD	83,3–100,0	100.0	100,0–100,0	100.0	0.280	m
SF-36 Vitality Score						
Pre LNG-IUD	30,0–65,0	50.0	35,0–56,3	45.0	0.970	m
Post LNG-IUD	47,5–78,0	60.0	45,0–75,0	60.0	0.454	m
SF-36 Mental Health Score						
Pre LNG-IUD	56,0–84,0	68.0	56,0–76,0	70.0	0.643	m
Post LNG-IUD	58,0–84,0	72.0	59,0–84,0	72.0	0.745	m
SF-36 Social Functioning						
Pre LNG-IUD	50,0–87,5	67.5	50,0–87,5	71.3	0.850	m
Post LNG-IUD	65,0–100,0	100.0	75,0–100,0	87.5	0.891	m
SF-36 Bodily Pain Score						
Pre LNG-IUD	45,0–100,0	80.0	45,0–100,0	93.8	0.953	m
Post LNG-IUD	83,8–100,0	100.0	75,0–100,0	100.0	0.715	m
SF-36 General Health						
Pre LNG-IUD	25,0–60,0	40.0	25,0–60,0	47.5	0.961	m
Post LNG-IUD	50,0–70,0	65.0	50,0–70,0	67.5	0.896	m
MMAS^§^ Total Score						
Pre LNG-IUD	30,3–68,1	48.9	30,2–73,7	42.5	0.781	m
Post LNG-IUD	77,2–100,0	100.0	80,2–100,0	100.0	0.850	m

Md: Median †: SF-36: 36-Item Short Form Health Survey ‡:LNG-IUD: The Levonorgestrel Releasing Intrauterine Device §:MMAS: Menorrhagia Multi-Attribute Scale.

## Discussion

In this study, endometrial pathology results obtained before LNG-IUD insertion were divided into two groups as normal cycle patterns and hormonal imbalance patterns. The results of the questionnaires were compared between the groups to evaluate the change in the quality of life of the patients after the use of LNG-IUD. It was found that there was no significant difference between the groups in MMAS scores, SF-36 scores, depression rates and treatment continuation rates (p > 0.05). In both groups, a significant increase in quality of life scores measured with SF-36 and MMAS was observed after LNG-IUD application (p < 0.05), and a significant decrease in Beck Depression Inventory scores and depression rates was recorded (p < 0.05). These findings strongly demonstrate the positive and consistent impact of LNG-IUD use, regardless of underlying endometrial pathology result.

In their retrospective study examining the endometrial pathology patterns seen in heavy menstrual bleeding, Vijayaraghavan A Sr et al. found normal cyclic endometrium in 56.9% of 160 cases. The most common pattern in our study was similarly normal cyclic endometrium (65%). The slightly higher incidence rate is due to the focus on the pathology results of patients undergoing LNG-IUD treatment, not all patients with heavy menstrual bleeding [10].

Husain et al. found that the prevalence of the most common endometrial pathology, the normal cyclic pattern, was higher in patients under 40 years old compared to those aged 40–55 (40.9% vs. 33%). Similarly, in our study, the age of patients in the normal cyclic pattern group was found to be significantly younger than that of patients in the hormonal imbalance group (41.1 ± 5.4 years vs. 44.1 ± 5.4 years p < 0.05) [11].

In retrospective studies by Agarwal et al., use of the LNG-IUD raised the mean MMAS score from 36.73 to 93.72 and statistical significantly improved SF-36 scores (p < 0.05). Continuation rate was 94.25% and hysterectomy rate after seven years was 5.75% [2]. In a study by Momoeda et al., MMAS median score rose from 47.7 to 89.3 at three months and 93.9 at twelve months. In our study, the MMAS score increased from 51.9 ± 27.8 to 83.8 ± 26.2 at six months, and SF-36 scores also improved statistically significantly (p < 0.05) [25]. Our LNG-IUD continuation rate was 83.2%, which is somewhat lower than that seen in Agarwal’s study.

Ishizawa C et al studied the outcome of LNG-IUD in patients with adenomyosis. They compared the MMAS score, hemoglobin level, and dysmenorrhea level (using the visual analog scale) after LNG-IUD insertion between early adenomyosis and advanced adenomyosis. Their results showed that LNG-IUD improved quality of life, hemoglobin levels, and dysmenorrhea more effectively in early adenomyosis. In this study, the effect of LNG-IUD treatment on quality of life was compared according to endometrial pathology results. A significant increase was seen in quality of life questionnaires in both groups, but no significant difference was found between the groups (p < 0.05) [17].

In the prospective, multi-center J-MIRAI study involving 47 cases that evaluated menstrual blood loss using the pictorial blood loss assessment chart (PBAC) and MMAS scores after LNG-IUD placement, they found that the MMAS score significantly increased from 50.50 before placement to 88.67 by the 12th month. This increase began after the third month and continued to rise until the twelfth month, aligning with the results of our study [26].

In the study by Zeliha Atak et al. involving 172 patients who received an LNG-IUD for abnormal uterine bleeding, 14% developed amenorrhea. Similarly, our study showed a 10.5% amenorrhea rate. Their removal rate was 15.1%, comparable to our 16.8% (including expulsions and voluntary removals) [27].

In the prospective study by Creinin MD et al., which included 105 patients with heavy menstrual bleeding, they measured menstrual products quantitatively at baseline, 3, and 6 months after LNG-IUD placement. They defined treatment success as having less than 80 mL of blood loss and at least a 50% reduction from baseline. They reported an expulsion rate of 13.3%, similar to our finding of 16.8%, and a treatment success rate of 81.8%. In contrast, our study evaluated treatment success based on improvements in quality of life and continuation rates, and 83.2% of participants reported being satisfied and continuing treatment [14].

In the survey by Matteson KA et al., comparing 29 LNG-IUD users and 33 patients on combined oral contraceptives, no significant difference in bleeding-related quality of life was found at 6 or 12 months [28]. In our study, quality of life after LNG-IUD use was compared by endometrial pathology. At 6 months, both SF-36 and MMAS scores improved significantly (p < 0.05) in all groups, but there were no statistically significant differences between pathology types.

Kavasoğlu et al. compared LNG-IUD and oral norethisterone acetate 10 mg in 192 patients with heavy menstrual bleeding and found depression in 1.2% of LNG-IUD users [29]. Similarly, Larsen SV et al. reported depression risks of 1.21% for low-dose, 1.46% for medium-dose, and 1.84% for high-dose LNG-IUDs, noting that higher doses slightly increased the risk of depression requiring treatment [30]. In contrast, no patients in our study developed depression after LNG-IUD insertion. Instead, a slight improvement was seen in Beck Depression Scale scores, possibly because LNG-IUD use in our study was for treating medical conditions rather than contraception, which may have improved quality of life.

The study was conducted retrospectively. Past symptoms may have been underestimated due to the memory factor of the cases. This is a limitation of the study. Another limitation of the study is that the Menorrhagia Multi-Attribute Scale (MMAS) used is a well-established and reliable instrument in the literature; however, a formal Turkish validation has not yet been conducted. However, to ensure clarity and comprehension, the questionnaire was administered in Turkish, and the consistency of responses—also aligning well with the SF-36 Health Survey results—suggests that this limitation is unlikely to have significantly influenced the overall reliability of the findings. Therefore, the results obtained from this questionnaire can be regarded as preliminary yet supporting data. A strength of the study is that it is the first to compare quality of life and treatment adherence rates between endometrial pathology groups prior to LNG-IUD application. Additionally, a statistically significant age difference between groups is noteworthy. Although this might initially be considered a potential confounding factor, it actually reinforces the study’s findings, demonstrating that despite differences in age and endometrial pathology patterns, the improvement in quality of life remained consistent across all groups. In clinical practice, these findings support the use of the LNG-IUS as a first-line treatment for all cases of benign endometrial pathology not requiring surgical intervention, as it consistently improves quality of life across different patient groups.

## Conclusion

This study makes a significant contribution to the literature as the first to comprehensively evaluate the impact of LNG-IUD on quality of life according to endometrial pathology results. It not only fills a critical gap in existing research but also provides valuable guidance for clinicians in selecting the most suitable patients for LNG-IUD treatment.

## Supporting information

S1 Data LNG IUD Patient Dataset. (XLSX)
